# P02.131. Effects of meditation on perceived stress, mood, sleep, memory and blood pressure in cognitively impaired adults and their caregivers: a pilot trial

**DOI:** 10.1186/1472-6882-12-S1-P187

**Published:** 2012-06-12

**Authors:** K Innes, T Selfe, C Brown, K Rose, A Thompson-Heisterman

**Affiliations:** 1 West Virginia University, Morgantown, USA; 2 JMU, Harrisonburg, USA; 3 University of Virginia, Charlottesville, USA

## Purpose

To investigate the effects of an 8-week meditation program on perceived stress, sleep, mood, sympathetic activation and memory function in adults with cognitive impairment and their caregivers.

## Methods

Six community-dwelling adults with an established diagnosis of mild cognitive impairment or early stage Alzheimer’s disease (4 women, 2 men), together with their live-in caregivers (3 women, 3 men), were enrolled in the study. Each participant dyad was trained in a basic Kundalini yoga meditation and asked to complete an 8-week meditation program (two 11-minute sessions per day) with the aid of an instruction sheet and meditation CD. Major outcomes included measures of stress (Perceived Stress Scale), sleep (General Sleep Disturbance Questionnaire), mood (Profile of Mood States), memory function (Memory Functioning Questionnaire), and sympathetic activation (blood pressure, heart rate). Participants were assessed at baseline and following completion of the 8-week program. Changes in specific measures over time (0-8 weeks) were analyzed using repeated measures ANOVA.

## Results

Ten participants (5 dyads) completed the study, including 6 women and 4 men. Compliance was very good overall, with participants completing an average of 11.4±1.1 meditation sessions/week. Although caregivers showed significantly greater sleep disturbance and superior memory functioning at baseline than did the cognitively impaired participants, treatment effects did not vary by participant status; analyses were thus pooled across participants. Participants demonstrated improvement in all major outcome measures following the 8-week intervention, including perceived stress (p<0.001), mood (overall, p=0.07; depression, p=0.01; anger, p=0.09), sleep quality (p<0.04), retrospective memory function (p=0.04), and blood pressure (systolic, p=0.004; diastolic, p=0.065).

## Conclusion

Findings of this exploratory trial suggest that an 8-week meditation program may offer an acceptable and effective intervention for reducing perceived stress and blood pressure, and improving certain domains of sleep, mood, and memory in adults with cognitive impairment and their caregivers.

